# Multidimensional evaluation of performance with experimental application of balanced scorecard: a two year experience

**DOI:** 10.1186/1478-7547-9-7

**Published:** 2011-05-17

**Authors:** Silvia Lupi, Adriano Verzola, Gianni Carandina, Manuela Salani, Paola Antonioli, Pasquale Gregorio

**Affiliations:** 1 Section of Hygiene and Occupational Medicine, Department of Clinical and Experimental Medicine, University of Ferrara, Italy; 2 Management Planning and Control St. Anna University Hospital, Ferrara, Italy; 3 Analysis Laboratory, St. Anna University Hospital, Ferrara, Italy; 4 Medical Direction Committee, St. Anna University Hospital, Ferrara, Italy

## Abstract

**Background:**

In today's dynamic health-care system, organizations such as hospitals are required to improve their performance for multiple stakeholders and deliver an integrated care that means to work effectively, be innovative and organize efficiently. Achieved goals and levels of quality can be successfully measured by a multidimensional approach like Balanced Scorecard (BSC). The aim of the study was to verify the opportunity to introduce BSC framework to measure performance in St. Anna University Hospital of Ferrara, applying it to the Clinical Laboratory Operative Unit in order to compare over time performance results and achievements of assigned targets.

**Methods:**

In the first experience with BSC we distinguished four perspectives, according to Kaplan and Norton, identified Key Performance Areas and Key Performance Indicators, set standards and weights for each objective, collected data for all indicators, recognized cause-and-effect relationships in a strategic map. One year later we proceeded with the next data collection and analysed the preservation of framework aptitude to measure Operative Unit performance. In addition, we verified the ability to underline links between strategic actions belonging to different perspectives in producing outcomes changes.

**Results:**

The BSC was found to be effective for underlining existing problems and identifying opportunities for improvements. The BSC also revealed the specific perspective contribution to overall performance enhancement. After time results comparison was possible depending on the selection of feasible and appropriate key performance indicators, which was occasionally limited by data collection problems.

**Conclusions:**

The first use of BSC to compare performance at Operative Unit level, in course of time, suggested this framework can be successfully adopted for results measuring and revealing effective health factors, allowing health-care quality improvements.

## Background

Health-care organizations are operating in a complex environment. Financial pressures from government, the need to arrange integrated care and improve performance for multiple stakeholders, as well escalating costs are driving administrators to search for effective management tools. In addition, all aspects of the sector are being asked to account for their performance and to demonstrate efficiency and effectiveness in providing services to their clients.

Financial measures alone are not sufficient to measure performance. Other factors missing from traditional financial reporting such as competence, customer focus, operational efficiency, innovation and knowledge must be carefully considered. Adopting Balanced Scorecard (BSC) in healthcare organization permits us to develop a more comprehensive set of performance indicators. The BSC is a management tool, originally applied to private sector, developed by Kaplan and Norton in 1992 [1]. Their framework broadened the traditional performance assessment approach by integrating financial measures with other key performance indicators linked to additional areas: customer preferences, internal business processes, organization growth, learning and development. Performance measures belonging to all four features are included in BSC [2].

About ten years after Kaplan and Norton developed BSC, a number of health-care organizations started to adapt and implement this framework in various settings from North America to Asia [3-5] and also in Europe [6,7] with the remarkable experience of NHS Performance Assessment Framework [8] in United Kingdom. In the past few years a growing number of Italian health-care institutions adopted BSC with the aim of measuring overall performance and to improve clinical and financial goals [9].

When applied to the health-care sector, the four traditional perspectives should be slightly modified to better display the functioning of public funded hospitals. The Financial Perspective should contain indicators of efficiency and asset utilization, including cost containment. Community Perspective should include measures of quality patient-centred care. Internal Processes Perspective should report indicators of continuous quality improvement and integrated service design. Growth and Learning Perspective should cover measures of human capital and strategic competencies. In each of the perspective significant success activities, indicated as Key Performance Areas (KPAs), are defined. Afterwards critical success factors, known as Key Performance Indicators (KPIs), are identified as well as measurement methods and standards. They balance between long term and short term in addition to internal and external factors contributing to business strategy that is translated into operational terms. Design of a strategic map, communicating outcomes to achieve by means of strategic initiatives for all Perspectives and their relationships, represents an essential component of BSC.

Traditionally financial metrics obtain increased importance than other parameters like quality of care, patient satisfaction, innovation, physicians and staff fulfillment.

In consequence of Laboratory Analysis management and staff requests for being evaluated, not only for financial outcomes, but also for relationships with community, internal procedures improvement, competence and knowledge, a first application of BSC was carried out with satisfactory results [10] in the past. In continuity with previous experience, the model was again applied, only with slight modifications to better depict Laboratory Analysis current activity. The objective of this paper is to confirm feasibility and value of using BSC to measure, over time, performance in Laboratory Analysis Operative Unit (OU) of St. Anna University Hospital, in particular the capacity to highlight outcome differences and explain their occurrence and relationships.

## Methods

We followed the methodological procedure established for precedent performance measuring by BSC. Briefly, as previously described [10], the major steps were:

• definition of strategic map divided into four Perspectives (Community, Internal Processes, Financial Resources, Growth and Learning) according Norton and Kaplan [1];

• identification of Key Performance Areas or macro-objectives, namely most important fields linked to abovementioned Perspectives in which not to fail [11];

• determination of cause/effect relationships between KPAs in order to explain interdependence among objectives belonging to different areas;

• description of pre-defined sub-objectives OU have to realize in order to accomplish KPAs;

• designation of Key Performance Indicators suitable for monitoring the degree of achievement of defined sub-objectives. In particular indicators that can effectively represent the phenomenon being measured were chosen from those reported in the text of Bocci and Miozzo [12] according to truth, focus, consistency, access, clarity, so what, timeliness, cost, gaming criteria as set by Neely and Kennerly [13].

• characterization of standard value (acceptable-expected value to obtain adequate quality of assistance) and weight (importance attributed to the indicator, highest sum of weights of each Perspective was equal to 100). Standard values were established in agreement with health workers analysing previous experience of OU. Associated weights were set up on the basis of mean weight assigned in order to permit balanced evaluation of OU performance and emphasize key-objectives by a pool of professionals belonging to assistance, organisational and directional fields. The adopted system of allocation of weights allowed us to understand areas and indicators, among those included in the evaluation, assuming greater importance for the organization depending on the business strategy.

• data collection;

• data ordering in spreadsheets.

Information has been drawn from various paper and digital sources. For example although St. Anna University Hospital does not have a computer platform dedicated to BSC, data is derived from SAP (cost containment, ticket collection), LIS (intra-lab reproducibility of results, activity indicators, external and internal TAT,) project SOLE database (number of GPs in the network, multi-typology of report receiving), quality indicators intranet database (MISA score), training office database (staff refresher courses).

At the second survey strategic map was confirmed. Minor changes were operated when chosen indicators were no longer detectable or available because of the ongoing transition of Laboratory Analysis in the unified Department with Ferrara Local Health Unit. In order to get an overall performance assessment for each Perspective, the weights of achieved goals were added and the obtained value was translated in a pictorial representation as traffic lights. Colour assigned in a summary table corresponding to prevalent assessment. Totally achieved goals are represented by green, completely not achieved goals are symbolized by red, while orange indicated a borderline condition due to an observed value slightly out of line with the fixed target or insurmountable difficulties which did not allow to reach the objective.

The first data collection partly referred to 2007 and partly to January-June 2008 because some indicators related to activities implemented at the beginning of 2008. The second data collection referred to the second part of 2008 and 2009.

On the basis of obtained data, a general evaluation panel of the entire Operative Unit was built, summarizing, for each Perspective, the achieved level of performance.

## Results

Data collection in two different surveys and Perspectives schedules completion permitted us to get an evaluation of performance trend over time. Results and a brief description of strategic map are shown below.

### Strategic map

Strategic map was built on four classical perspectives identified by Kaplan and Norton [1] with the exception of Community Perspective that was defined as an area including objectives linked to different stakeholders: Users such as as patients, hospital doctors, general practitioners; Owners of public healthcare services; Public entities including laws that protect community and environment. Internal Procedures Perspective referred to how specific processes are performed, including service appropriateness and innovation, relationship with users, quality of hygiene and organisational standards, risk management, accreditation. To ensure financial sustainability was identified as main objective in Financial Resources Perspective, taking into account the urgent need to ensure the financial stability of public healthcare hospital. For Growth and Learning Perspective we considered staff continuous updating, improvement of computer infrastructure supporting informative flows, organisational resources as team-work, leadership, alignment to organisation strategy. Objectives have been identified trying to keep in mind the link between them and different perspectives. The map was drawn up in the belief that it can be a valuable tool allowing the reading of close cause-effect relationships between various strategic objectives and enabling their accomplishment. Review at the second survey confirmed the structure previously outlined that is shown in Additional File 1.

### Community Perspective

In this Perspective (Additional File 2), six of the assigned objectives were fully met (score 57 out 100), while two presented a borderline condition and two others showed a misalignment highlighted by red colour. Compared to first survey, some conditions have changed: we reported an improvement in providing timely responses to emergency requests, moving from a critical situation to a value slightly below the standard set, but also a worsening due to increase of complaints.

The overall Perspective assessment, with recorded improvements, described a positive trend therefore pictorial representation has changed from orange to green.

### Internal Processes Perspective

Almost all given objectives resulted in achieved levels and were therefore marked by a green signal (Additional File 3). The score was 65 out of 95 because a goal with weight 5 could not be evaluated and was postponed to next year. We showed a reduction in the average number of withdrawals per operator and a trend to alignment for optimization of urgent analysis procedures. Both conditions are marked with orange. General evaluation was wholly satisfactory and then characterized by green colour confirming a positive result of the first survey.

### Financial Resources Perspective

Objectives assessed were almost totally aligned with set standards (Additional File 4) with a score of 75 out of 100. The global level of performance is therefore still marked, also for the second detection, with a green signal.

### Growth and Learning Perspective

Allocated goals were entirely achieved reaching a score of 70 out of 70 (Additional File 5) because the objective related to operators' satisfaction and could not be evaluated since no further organisational wellbeing survey has been conducted. Positive green indication, as obtained in first survey, was confirmed.

### Overall evaluation

Comparison between the two surveys, reported in summary table (Additional File 6), confirmed a fully satisfactory performance highlighted by green colour for all considered aspects. Particularly overtime evaluation pointed out improvement reached in Community Perspective.

## Discussion

Application of experimental model allowed to build a summary table showing changes in performance according to selective Perspectives. In the first survey, Community Perspective obtained an orange alert signal indicating a situation of partial mismatch for some of its objectives, as other Perspectives showed a complete achievement of fixed goals. While Internal Processes, Financial Resources and Growth and Learning Perspectives resulted unmodified, confirming a positive performance, some improvements have changed Community Perspective situation leading to a positive signal in the second survey.

Reasons that led to this final score can be better highlighted considering in detail specific tables. Maintaining the positive performance was not simply due to a stable condition but several improvements were noticed. Particularly, for Community Perspective, we found enhanced ability to provide a timely response to emergency requests, an increased number of reports delivered by web, reduction of waiting times for access to appointments. However, there are also some deteriorations, such as a lack of identification badge for staff in contact with patients and the rising of complaints (mainly due to changes in set of rules for payment). Also other Perspectives confirmed positive results due to the maintenance of capacity to achieve the objectives and further improvement efforts, including better analytical accuracy, increased number of GPs becoming web connected, largest commitment of staff in teaching activities and tutoring.

The tested and adopted BSC model proved to be useful in highlighting variations by changing colours in pictorial representation for Community Perspective. Actually this Perspective experienced greatest ferment situation characterized by improvements and worsening. Furthermore, the model allowed us to get critical issues of an indicator, its impact on the area to which it belongs, allowing analysis, maintaining the indicator controlled and preparing corrective actions.

Applying our experimental model of BSC, we were interested to test the understanding of interdependence relations between the different Perspectives to confirm the assumptions on which construction of Strategic Map, indicative of the strategy is based [14,15]. In detail, as showed in Additional File 1 and illustrated by blue arrows, the improvement in information technology in Growth and Learning Perspective, with increased number of web connected GPs, has led to an augmented number of reports delivered by web. This issue was included in innovation in service production and offer, a KPA of Internal Processes Perspective that can be connected to service appropriateness and meeting the health needs of the population in collaboration with other operators, KPAs of Community Perspective. It is reasonable to assume an upcoming positive influence on business synergies and economic efficiency, KPAs belonging to Financial Resources Perspective.

In reference to the two observed worsening situations, they were not shown by pictorial representation probably because these indicators have been assigned a low weight. A further explanation could be related to the type of reporting chosen, that is based on three levels, while adoption of a system more appropriate to emphasize borderline situations could solve this problem.

## Conclusions

As exposed in previous work [10], the experienced BSC model showed strengths and weaknesses, however it was found to be effective for underlining existing problems and identifying opportunities for improvement, as confirmed in this paper. In addition we assessed the ability to capture connections of measured results to strategy and their cause-and-effect linkages that describe the hypotheses of the strategy [16]. Main difficulties lie in choosing appropriate indicators and the subsequent assignment of weights, avoiding under or over estimation, and standards. Preference must be calibrated according to specific situation and must not allow generalizations to better describe context of reference.

Furthermore performance comparisons using the BSC depend on selection of feasible and appropriate Key Performance Indicators, which is occasionally limited by data collection problems, for example, constant updating to adapt to evolving context changes may impose KPI variations, inducing the lack of reference to historical data.

BSC was an ideal point of contact between clinical and economic dimension and allowed us to perceive improved results as a consequence of progress in different inter-related perspectives.

## Abbreviations

Balanced Scorecard: BSC; Coefficient of Variation: CV; General Practitioner: GP; Key Performance Area: KPA; Key Performance Indicator: KPI; Laboratory Information System: LIS; Mean Index of Deviation: MISA; Mixed Advisory Committee: MAC; Operative Unit: OU; Oral Anticoagulant Therapy: OAT; Sanità On LinE (e-Health): SOLE; Time Around Time: TAT; Unique Appointment Centre: UAC.

## Competing interests

The authors declare that they have no competing interests.

## Authors' contributions

AV, GC and PG conceived the study, participated in study design and coordination. SL, AV, MS, and PA performed acquisition, analysis and interpretation of data. AV and SL drafted the manuscript. All authors read and approved the final manuscript.

## Supplementary Material

Additional file 1 **Strategic Map_Additional file 1**. The file contains a strategic map of Laboratory Analysis in which we highlighted links between KPIs of different KPAs, as emerged from results of the two surveys, that have led to an improvement found in the overall performance of Operative Unit. Click here for file

Additional file 2 **Community Perspective Table_Additional file 2**. The file contains a table resuming macro- and specific objectives referring to KPAs, indicators and standards referring to KPIs, results obtained in the two different observations of Community Perspective. Click here for file

Additional file 3 **Internal Processes Perspective Table_Additional file 3**. The file contains a table resuming macro- and specific objectives referring to KPAs, indicators and standards referring to KPIs, results obtained in the two different observations of Internal Processes Perspective. Click here for file

Additional file 4 **Financial Resources Perspective Table_Additional file 4**. The file contains a table resuming macro- and specific objectives referring to KPAs, indicators and standards referring to KPIs, results obtained in the two different observations of Financial Resources Perspective. Click here for file

Additional file 5 **Growth and Learning Perspective Table_Additional file 5**. The file contains a table resuming macro- and specific objectives referring to KPAs, indicators and standards referring to KPIs, results obtained in the two different observations of Growth and Learning Perspective. Click here for file

Additional file 6 **Global Performance Table_Additional file 6**. The file contains a table resuming global performance reached in all four Perspectives. Click here for file

## References

[B1] Kaplan RS Norton DP The Balanced Scorecard: measures that drive performance Harvard Business Review 1992 Jan-Feb 71 79 10119714

[B2] Kaplan RS Norton DP Using the Balanced Scorecard as a strategic management system Harvard Business Review 1996 Jan-Feb 75 85

[B3] Parkinson J Tsasis P Porporato M A critical review of financial measures as reported in the Ontario hospital balanced scorecard J Health Care Finance 2007 34 2 48 56 18972993

[B4] Young J Bell R Khalfan A Lindquist E Evaluating the balanced scorecard at the University Health Network: an impact assessment Healthc Q 2008 11 2 52 56 18362520 10.12927/hcq.2008.19616

[B5] Chen XY Yamauchi K Kato K Nishimura A Ito K Using the balanced scorecard to measure Chinese and Japanese hospital performance Int J Health Care Qual Assur Inc Leadersh Health Serv 2006 19 4-5 339 350 16961099 10.1108/09526860610671391

[B6] Ten Asbroek AHA Arah OA Geelhoed J Custers T Delnoij DM Klazinga S Developing a national performance indicator framework for the Dutch health system Int J Qual in Health Care 2004 16 Supplement 1 i65 i71 10.1093/intqhc/mzh020 15059989

[B7] Gurd B Gao T Lives in the balance: an analysis of the balanced scorecard (BSC) in healthcare organizations Int J Productivity Performance Management 2008 57 1 6 21 10.1108/17410400810841209

[B8] Chang L The NHS performance assessment framework as a balanced scorecard approach: Limitations and implications Int J Public Sector Management 2007 20 2 101 117 10.1108/09513550710731472

[B9] Baraldi S Il Balanced Scorecard nelle aziende sanitarie 2005 Mc Graw-Hill; Milano

[B10] Verzola A Bentivegna R Carandina G Trevisani L Gregorio P Mandini A Multidimensional evaluation of performance: experimental application of the Balanced Scorecard in Ferrara university hospital Cost Eff Resour Alloc 2009 7 15 http://www.resource-allocation.com/content/7/1/15 10.1186/1478-7547-7-15 PMC2759901 19737409

[B11] Fioravanti L Spadonaro F Una valutazione multidimensionale della performance dei Sistemi Sanitari Nazionali Sanità Pubblica e Privata 2005 4 30 4021137027

[B12] Bocci F Miozzo A La Balanced Scorecard orientata alla mission 2006 Il Sole 24 ore; Milano

[B13] Kennerley M Neely A Measuring performance in a changing business environment International Journal of Operations & Production Management 2003 23 213 229 10.1108/01443570310458465

[B14] Kaplan RS Norton DP The Balanced Scorecard 1996 Harvard Business School Press; Boston 10119714

[B15] Kaplan RS Norton DP Having trouble with your strategy? Then map it Harvard Business Review 2000 Sept-Oct 167 176 11143152

[B16] Kaplan RS Norton DP Transforming the BSC from performance measurement to strategic management: part I Accounting Horizons 2001 March 87 104

