# Supplementary material for: Multidimensional evaluation of performance with experimental application of balanced scorecard: a two year experience
Source: Cost Eff Resour Alloc. 2011 May 17;9:7. doi: 10.1186/1478-7547-9-7 (PMC3118336; doi:10.1186/1478-7547-9-7)
Supplement: Additional file 1 — Strategic Map_Additional file 1. The file contains a strategic map of Laboratory Analysis in which we highlighted links between KPIs of different KPAs, as emerged from results of the two surveys, that have led to an improvement found in the overall performance of Operative Unit. [file 1478-7547-9-7-S1.PDF]

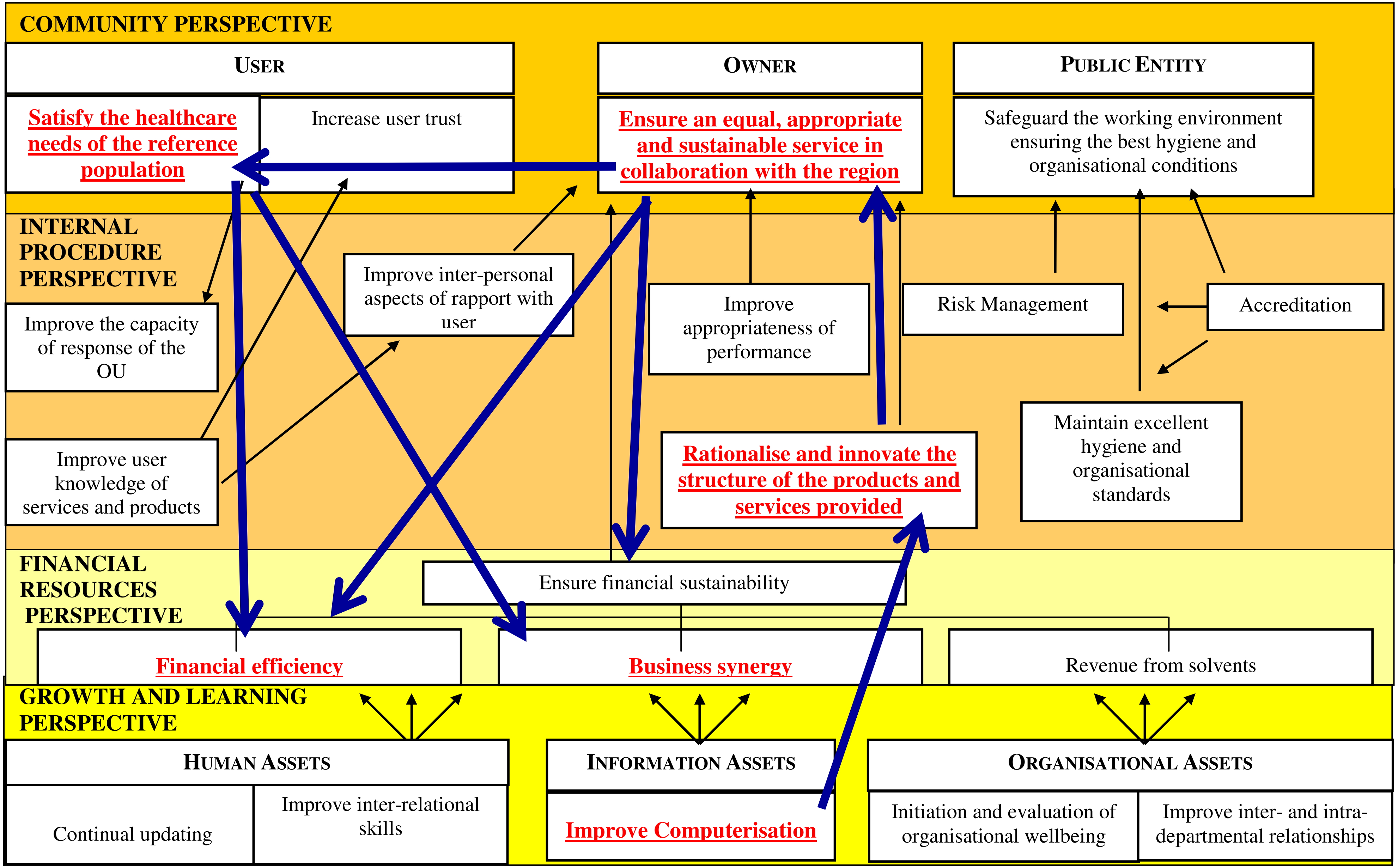
COMMUNITY PERSPECTIVE
USER
Satisfy the healthcare needs of the reference population
Increase user trust
OWNER
Ensure an equal, appropriate and sustainable service in collaboration with the region
PUBLIC ENTITY
Safeguard the working environment ensuring the best hygiene and organisational conditions
INTERNAL PROCEDURE PERSPECTIVE
Improve the capacity of response of the OU
Improve inter-personal aspects of rapport with user
Improve appropriateness of performance
Risk Management
Accreditation
Improve user knowledge of services and products
Rationalise and innovate the structure of the products and services provided
Maintain excellent hygiene and organisational standards
FINANCIAL RESOURCES PERSPECTIVE
Ensure financial sustainability
Financial efficiency
Business synergy
Revenue from solvents
GROWTH AND LEARNING PERSPECTIVE
HUMAN ASSETS
Continual updating
Improve inter-relational skills
INFORMATION ASSETS
Improve Computerisation
ORGANISATIONAL ASSETS
Initiation and evaluation of organisational wellbeing
Improve inter- and intra-departmental relationships

STRATEGIC MAP

As reported in the previous paper [10], the Strategic Map specifies objectives and interconnections running through the four Perspectives. Repeated detection of achieved results for KPIs permitted to verify cause-and-effect linkages that were previously only hypothesized. In particular, links shown by blue arrows could be identified. Rise in number of web connected GPs, indicator belonging to improvement in information technology KPA of Growth and Learning Perspective allowed to deliver more reports by web, improving innovation in service production and offer, a KPA of Internal Processes Perspective. In Community Perspective, this relates to service appropriateness and subsequently to meeting health needs of the population in collaboration with other operators. Attainment of these two objectives can improve business synergies and economic efficiency, KPAs belonging to Financial Resources Perspective.
