# Supplementary material for: Multidimensional evaluation of performance with experimental application of balanced scorecard: a two year experience
Source: Cost Eff Resour Alloc. 2011 May 17;9:7. doi: 10.1186/1478-7547-9-7 (PMC3118336; doi:10.1186/1478-7547-9-7)
Supplement: Additional file 2 — Community Perspective Table_Additional file 2. The file contains a table resuming macro- and specific objectives referring to KPAs, indicators and standards referring to KPIs, results obtained in the two different observations of Community Perspective. [file 1478-7547-9-7-S2.PDF]

Macro-Objective	Specific Objective	Indicator	Weight	Standard	First observation[a]		Second observation[b]	
					Observed value	Pictorial representation	Observed value	Pictorial representation
**USER**<br><br>Satisfy healthcare needs of reference population	Increase user trust	N° of complaints	20	< 2 complaints/year	2007: 3 complaints	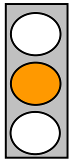	2008: 6 complaints	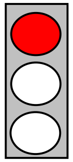
	Timeliness	Internal turn-around time (TAT):<br>1) Emergencies: TAT <30 min./total emergency requests<br>2) Urgencies: TAT <2 h/total urgent requests<br>3) Urgencies/emergencies: TAT< 1 h total urgent-emergency requests*	12	1) ≥70%<br>2) ≥90%<br>3) ≥90%*	1) 8.1%<br>2) 38.1%<br>(July-September 2008)	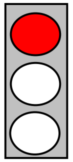	*3) 86.4%<br>(July 2008- June 2009)	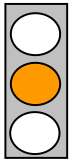
		External turn-around time (TAT):<br>1) Web OAT: TAT by 12.30 a.m. of the following day/total Web OAT requests<br>2) SOLE: TAT within 1 day of conclusion /total requests	12	≥95%	1) 97.3%<br>2) 100%<br>(January-July 2008)	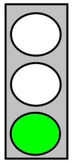	1) 98.0%<br>2) 100%<br>(July 2008- June 2009)	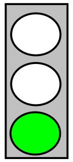
	Convenience	Multi typology of report receiving: (direct delivery, by mail, by web)<br>1) Number of reports delivered by mail/total reports<br>2) Number of reports delivered by SOLE (where activated)<br>3) Number of reports delivered by Web OAT (where available)	10	1) ≥5%<br>2) ≥3.00<br>3) ≥1.000	1) 1.86%<br>2) 3.462<br>3) 1.658<br>(January-April 2008)	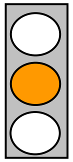	1) 7.82%<br>2) 14.927<br>3) 3.788<br>(July - December 2008)	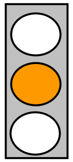
		Automated withdrawal points in Central Health Structures	5	100%	100% (2008)	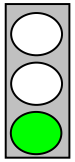	100% (2009)	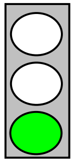

Macro-Objective	Specific Objective	Indicator	Weight	Standard	First observation[a]		Second observation[b]	
					Observed value	Pictorial representation	Observed value	Pictorial representation
**OWNER**<br><br>Ensure an equal, appropriate and sustainable service in collaboration with region	Budget result	Final balance in line with estimated budget	6	Alignment	Difference: 5.4% (2007)	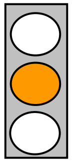	Difference: 9.5% (January-June 2009)	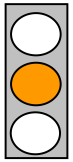
**PUBLIC ENTITY**<br><br>Safeguard the working environment ensuring the best hygiene and organisational conditions	Access easiness	Wait for access/working days	20	Appointments:≤5 working days facilitated categories: free access without appointment ≥10% for 90% of working days	appointments: 5.4 days facilitated categories: acceptable (January-July 2008)	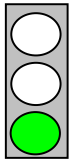	appointments: 3.11 days facilitated categories: acceptable (2009)	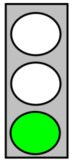
		Appointment Points	5	UAC list and Pharmacy with UAC function: 100%	100% (2008)	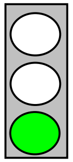	100% (2009)	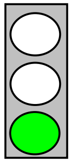
		Open blood collection points/total blood collection points	5	1) Working days: 100%<br>2) Saturday: at least 1 blood collection point per district	1) 100%<br>2) acceptable (January-June 2008)	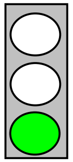	1) 100%<br>2) acceptable (2009)	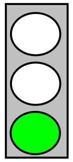
	Transparency	Employees in contact with users wearing ID badges/total employees	5	≥95% of employees	100% (2007)	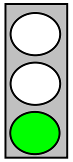	0% (2008)	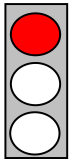

*only for second observation
[a] First data collection partly referred to 2007 and partly to January-June 2008 because some indicators related to activities implemented at the beginning of 2008.
[b] Second data collection referred to second part of 2008 and 2009.

COMMUNITY PERSPECTIVE

In this Perspective, objectives, standards, assigned weights, manner and frequency of data acquisition were the same as described in the previous paper [10] with a slight modification for internal Time Around Time (TAT) indicator of Timeliness KPA. Two different indicators, no longer available, were identified while in current survey emergencies and urgencies have been grouped into a single one, because of the ongoing transition of Laboratory Analysis into the unified Department with Ferrara Local Health Unit. For second observation they are replaced by Urgencies/emergencies with TAT< 1 h/total urgent-emergency requests. Standard was (defined in agreement with health workers on the basis of past experience) ≥90%. Weight was 12%, manner of detection was a verification carried out by the Analysis Laboratory and frequency of acquisition was every three months.
