# Supplementary material for: Multidimensional evaluation of performance with experimental application of balanced scorecard: a two year experience
Source: Cost Eff Resour Alloc. 2011 May 17;9:7. doi: 10.1186/1478-7547-9-7 (PMC3118336; doi:10.1186/1478-7547-9-7)
Supplement: Additional file 3 — Internal Processes Perspective Table_Additional file 3. The file contains a table resuming macro- and specific objectives referring to KPAs, indicators and standards referring to KPIs, results obtained in the two different observations of Internal Processes Perspective. [file 1478-7547-9-7-S3.PDF]

Macro-Objective	Specific Objective	Indicator	Weight	Standard	First observation[a]		Second observation[b]	
					Observed value	Pictorial representation	Observed value	Pictorial representation
Rationalise and innovate the structure of the products and services<br><br>Improve user knowledge of services and products provided<br><br>Improve the capacity of response of the OU	Constitution of integrated H&S network	Number and type of integrations and agreements with other health agencies/year	10	≥6	10 (2007)	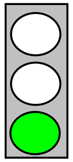	9 (2008)	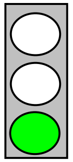
	Improvement of appointments service	Number of GPs in the network (SOLE- Web OAT)/GPs in central-northern district of Ferrara	5	≥30%	46.4% (2008)	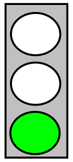	88.0% (2009)	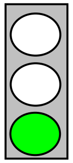
		Appointment reports/free access: samples in free access/total samples	5	≥10%	19% (January-June 2008)	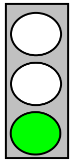	20% (2009)	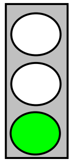
	Optimisation of acceptance and sampling procedure	Mean samples per operator/premises/hour	16	≥22/hour	23/hour (January-June 2008)	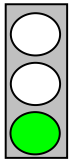	21/hour (2009)	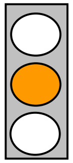
	Optimisation of Analysis and Validation Report/quality procedure	Intra-lab reproducibility of results (CV%)	8	<5% for 80% of chemical/clinical tests; <10% for 50% of immunometric tests	All values are in the range (January-June 2008)	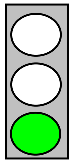	All values are in the range (2009)	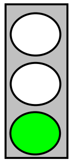
		Responsibility grid: functional units applying grid/total functional units	7	100%	100% (2007)	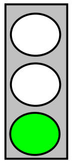	100% (2009)	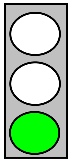

Macro-Objective	Specific Objective	Indicator	Weight	Standard	First observation[a]		Second observation[b]	
					Observed value	Pictorial representation	Observed value	Pictorial representation
Risk Management	Sentinel Events: reporting Sentinel Events	Specific reports of OU Manager on actions carried out	5	Evidence of reports	The specific reports have been produced (2007)	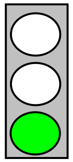	The specific objective could not be evaluated (2008) and it has been postponed until next year	
Improve inter-personal aspects of rapport with user	Maintain levels of communication	Verification by MAC of information sheet provision upon admission	5	Report of MAC	MAC report has been produced (2007)	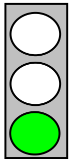	MAC report has been produced (2008)	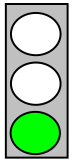
Accreditation	Prepare and maintain accreditation	Internal inspection and update documentation	10	Evidence of internal inspection passed and updated documents	Evidence of internal inspection passed and updated documents have been produced (2007)	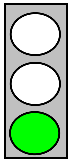	Evidence of internal inspection passed and updated documents have been produced (2009)	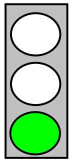
Improve appropriateness of performance	Chemical and clinical analytical accuracy	MISA score (indicator of performance: global evaluation of Laboratory)	15	≤100 (50-100=acceptable performance; <50 good performance)	81 (2007)	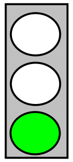	58 (2008)	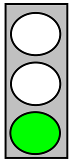
	Optimisation of urgent analysis procedure	Internal turn-around time (TAT) for Urgencies: 1) TAT <2 h/total urgent requests 2) TAT< 1 h total urgent-emergency requests*	14	1) ≥90% 2) ≥90%*	1) 38.1% (July-September 2008)	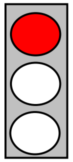	*2) 86.4% (July 2008- June 2009)	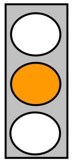

*only for second observation

[a] First data collection partly referred to 2007 and partly to January-June 2008 because some indicators related to activities implemented at the beginning of 2008.

[b] Second data collection referred to second part of 2008 and 2009.

INTERNAL PROCESSES PERSPECTIVE

Objectives, standards, assigned weights, manner and frequency of data acquisition were maintained as described in previous paper [10] with slight adaptations.

Regarding the objective of optimisation and validation of analysis, inter-laboratory comparability indicator was eliminated because afterwards included in MISA score. The corresponding weight was divided and assigned to applying responsibility grid and intra-lab reproducibility of results that assumed value of 7 and 8 respectively

About Risk Management, reporting sentinel events objective could not be evaluated in present analysis because it has been postponed to next year.

As reported for Community Perspective, internal Time Around Time (TAT) for Urgencies was no longer available, and urgencies have been defined in the current survey by a new indicator, because of the ongoing transition of Laboratory Analysis into the unified Department with Ferrara Local Health Unit. For second observation it is replaced by Urgencies/emergencies with TAT< 1 h/total urgent-emergency requests. Standard was (defined in agreement with health workers on the basis of past experience) ≥90%. Weight was 12%, the manner of detection was a verification carried out by the Analysis Laboratory and frequency of acquisition was every three months.
