# Supplementary material for: Multidimensional evaluation of performance with experimental application of balanced scorecard: a two year experience
Source: Cost Eff Resour Alloc. 2011 May 17;9:7. doi: 10.1186/1478-7547-9-7 (PMC3118336; doi:10.1186/1478-7547-9-7)
Supplement: Additional file 4 — Financial Resources Perspective Table_Additional file 4. The file contains a table resuming macro- and specific objectives referring to KPAs, indicators and standards referring to KPIs, results obtained in the two different observations of Financial Resources Perspective. [file 1478-7547-9-7-S4.PDF]

Macro-Objective	Specific Objective	Indicator	Weight	Standard	First observation[a]		Second observation[b]	
					Observed value	Pictorial representation	Observed value	Pictorial representation
**Ensure financial sustainability**	Maintain Provincial production levels	Report of production	20	Current production value equal to previous year's production value	2006: 4.947.668<br>2007: 5.423.331	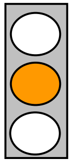	2008: 5.268.418	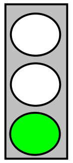
	Cost containment action	Comparison report of production/contribution margin	25	Current value equal to previous year value	2006: 2.356.474<br>2007: 3.252.614	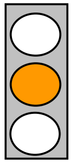	2008: 4.240.439	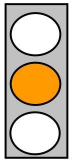
	Check budget adherence for Pharmacy and Bursary	Quarterly report comparing real and expected expenditure	30	Alignment	2006: 4.027.088<br>2007: 4.041.503	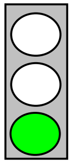	2008: 4.217.955	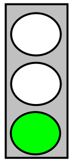
	Check cost report for expenditure/revenue	On-line report	20	Current value less or equal to previous year value	2006:67.64%<br>2007:60.52%	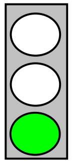	2008: 54,02%	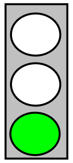
	Business synergy	Assets from ticket taking	5	>1.800.000 €/year	1.162.396 (January-July 2008)	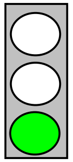	2009: 2.113.186	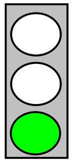

[a] First data collection partly referred to 2007 and partly to January-June 2008 because some indicators related to activities implemented at the beginning of 2008.
[b] Second data collection referred to second part of 2008 and 2009.

FINANCIAL RESOURCES PERSPECTIVE

Objectives, standards, assigned weights, manner and frequency of data acquisition were maintained unchanged as compared to previous survey [10].
