# Supplementary material for: Multidimensional evaluation of performance with experimental application of balanced scorecard: a two year experience
Source: Cost Eff Resour Alloc. 2011 May 17;9:7. doi: 10.1186/1478-7547-9-7 (PMC3118336; doi:10.1186/1478-7547-9-7)
Supplement: Additional file 5 — Growth and Learning Perspective Table_Additional file 5. The file contains a table resuming macro- and specific objectives referring to KPAs, indicators and standards referring to KPIs, results obtained in the two different observations of Growth and Learning Perspective. [file 1478-7547-9-7-S5.PDF]

Macro-Objective	Specific Objective	Indicator	Weight	Standard	First observation[a]		Second observation[b]	
					Observed value	Pictorial representation	Observed value	Pictorial representation
**Human Assets**<br><br>Continual updating<br><br>Improve inter-relational skills	Refresher courses for all staff members	Number of credits/year/employee	15	≥30	2007: Mean number of credits / employee > 30	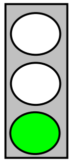	2008: Mean number of credits / employee > 30	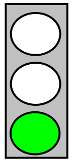
	Improve interpersonal skills	Number of complaints regarding interpersonal skills	10	0	2007: 0	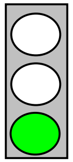	2008:0	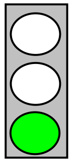
	Teaching and research activity	Participation in research programmes/year	20	≥1	2007: 1	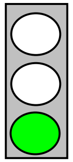	2008: 2	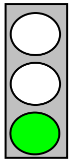
		Teaching and tutoring activity	20	OU staff involvement in teaching in at least 10 courses	2007: 7	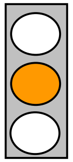	2008: 10 courses, 6 tutors	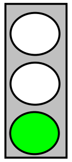
**Organisational Assets**<br><br>Evaluation of organisational wellbeing<br>Improve inter- and intra-departmental relationships	Satisfaction of operators/ Organisational wellbeing	Organisational wellbeing questionnaire	30	Current mean detection value of organisational wellbeing > previous value	2004: 5.21<br>2001: 4.52	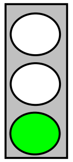	Objective not evaluated	

Macro-Objective	Specific Objective	Indicator	Weight	Standard	First observation[a]		Second observation[b]	
					Observed value	Pictorial representation	Observed value	Pictorial representation
**Information Assets**<br><br>Improve Information Communication Technology	Information Technology	Number of web connected GPs (SOLE/Web OAT)/ GPs in central-northern district of Ferrara	5	≥30%	2008: 46.4%	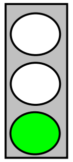	2009: 88%	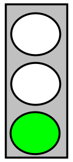

[a] First data collection partly referred to 2007 and partly to January-June 2008 because some indicators related to activities implemented at the beginning of 2008.
[b] Second data collection referred to second part of 2008 and 2009.

Growth and Learning Perspective

Objectives, standards, assigned weights, manner and frequency of data acquisition were maintained as described in previous paper [10], with the exception of the assessment of operators' satisfaction that could not be evaluated because no further surveys have been conducted.
