# Supplementary material for: Multidimensional evaluation of performance with experimental application of balanced scorecard: a two year experience
Source: Cost Eff Resour Alloc. 2011 May 17;9:7. doi: 10.1186/1478-7547-9-7 (PMC3118336; doi:10.1186/1478-7547-9-7)
Supplement: Additional file 6 — Global Performance Table_Additional file 6. The file contains a table resuming global performance reached in all four Perspectives. [file 1478-7547-9-7-S6.PDF]

Perspective	First observation[a]	Second observation[b]
COMMUNITY	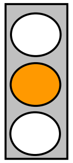	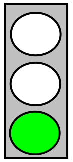
INTERNAL PROCESSES	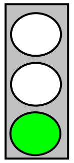	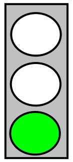
FINANCIAL RESOURCES	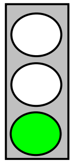	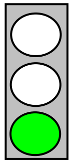
GROWTH AND LEARNING	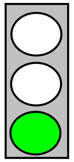	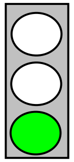

Summarizing evaluation panel of Laboratory Analysis performance obtained in the two periods of the survey.

[a] First data collection partly referred to 2007 and partly to January-June 2008 because some indicators related to activities implemented at the beginning of 2008.

[b] Second data collection referred to second part of 2008 and 2009.
